# Whole genome sequencing of amplified *Plasmodium knowlesi* DNA from unprocessed blood reveals genetic exchange events between Malaysian Peninsular and Borneo subpopulations

**DOI:** 10.1038/s41598-019-46398-z

**Published:** 2019-07-08

**Authors:** Ernest Diez Benavente, Ana Rita Gomes, Jeremy Ryan De Silva, Matthew Grigg, Harriet Walker, Bridget E. Barber, Timothy William, Tsin Wen Yeo, Paola Florez de Sessions, Abhinay Ramaprasad, Amy Ibrahim, James Charleston, Martin L. Hibberd, Arnab Pain, Robert W. Moon, Sarah Auburn, Lau Yee Ling, Nicholas M. Anstey, Taane G. Clark, Susana Campino

**Affiliations:** 1 Faculty of Infectious and Tropical Diseases, London School of Hygiene and Tropical Medicine, London, United Kingdom; 2 Centre Hospitalier Universitaire de Montpellier, Montpellier, France; 3 University of Malaya, Kuala Lumpur, Malaysia; 4 Global and Tropical Health Division, Menzies School of Health Research and Charles Darwin University, Darwin, Northern Territory Australia; 5 Infectious Diseases Society Sabah-Menzies School of Health Research Clinical Research Unit, 88300 Kota Kinabalu, Sabah Malaysia; 6 Clinical Research Centre, Queen Elizabeth Hospital, 88300 Kota Kinabalu, Sabah Malaysia; 7 Jesselton Medical Centre, 88300 Kota Kinabalu, Sabah Malaysia; 8 Genomics Institute, Biopolis, Singapore; 9 King Abdullah University of Science and Technology, Thuwal, Saudi Arabia; 10 Faculty of Epidemiology and Population Health, London School of Hygiene and Tropical Medicine, London, United Kingdom

**Keywords:** Bioinformatics, Genomic analysis, Genomics

## Abstract

The zoonotic *Plasmodium knowlesi* parasite is the most common cause of human malaria in Malaysia. Genetic analysis has shown that the parasites are divided into three subpopulations according to their geographic origin (Peninsular or Borneo) and, in Borneo, their macaque host (*Macaca fascicularis* or *M*. *nemestrina*). Whilst evidence suggests that genetic exchange events have occurred between the two Borneo subpopulations, the picture is unclear in less studied Peninsular strains. One difficulty is that *P*. *knowlesi* infected individuals tend to present with low parasitaemia leading to samples with insufficient DNA for whole genome sequencing. Here, using a parasite selective whole genome amplification approach on unprocessed blood samples, we were able to analyse recent genomes sourced from both Peninsular Malaysia and Borneo. The analysis provides evidence that recombination events are present in the Peninsular Malaysia parasite subpopulation, which have acquired fragments of the *M*. *nemestrina* associated subpopulation genotype, including the *DBPβ* and *NBPXa* erythrocyte invasion genes. The *NBPXb* invasion gene has also been exchanged within the macaque host-associated subpopulations of Malaysian Borneo. Our work provides strong evidence that exchange events are far more ubiquitous than expected and should be taken into consideration when studying the highly complex *P*. *knowlesi* population structure.

## Introduction

*Plasmodium knowlesi*, a common malaria parasite of long-tailed (*Macaca fascicularis*) and pig-tailed (*M*. *nemestrina*) macaques, is now recognized as a significant cause of human malaria, with cases reported across all countries of Southeast Asia^1–4^. *P*. *knowlesi* is the predominant cause of malaria in Malaysia^1–4^. The *Plasmodium* species can be transmitted through several vector species, including *Anopheles latens* and *A*. *balbacensis* in Malaysian Borneo^5–7^ and *A*. *hackeri* and *A*. *cracens* in Peninsular Malaysia^8^. Severe disease occurs in 6–9% of clinical presentations and fatalities have been described^1,9,10^. Rapid human population growth and deforestation, which can drive both encroachment on wild macaque habitats and vector distribution changes^11^, are thought to increase human-macaque contact, change transmission dynamics, and drive up the incidence of human *P*. *knowlesi* infections^12,13^. Regional elimination efforts have targeted *P*. *falciparum* and *P*. *vivax* transmission, with significant progress being demonstrated by the near-elimination of these *Plasmodium* species from areas such as Malaysian Borneo^3,4,14^. However, due to the difficulties in reducing the monkey parasite reservoir, it is unclear if similar control approaches are able to limit the risk of humans acquiring *P*. *knowlesi* malaria^15,16^.

Appropriate molecular tools and sampling are needed to assist surveillance of *P*. *knowlesi* by malaria control programs, and to understand its genetic diversity and transmission. *P*. *knowlesi* genomics could lead to biological insights that inform control measures. Advances in whole genome sequencing (WGS) technologies have led to the characterization of single nucleotide polymorphisms (SNPs) across *P*. *falciparum* and *P*. *vivax*, with an improved understanding of their population structure and diversity, as well as loci underpinning drug resistance (e.g.^17–22^). For *P*. *knowlesi* WGS studies, the number of high quality isolates analysed in each study has been small (n < 70)^23–25^. However, these studies have revealed that the *P*. *knowlesi* genome is more polymorphic than *P*. *falciparum*, and that three main subpopulations exist based on geographical source (Peninsular-Malaysia vs. Malaysian Borneo) and, within Malaysian Borneo, different hosts (*M*. *nemestrina* [*Mn-Pk*] and *M*. *fascicularis* [*Mf-Pk*] macaques, and humans)^23–25^. These studies have also provided evidence that *P*. *knowlesi* nuclear genomes are not genetically isolated, and there have been chromosomal-segment exchanges between subpopulations^23–25^. This observation points to subpopulations that have diverged in isolation and then re-connected, possibly due to deforestation and disruption of wild macaque habitats^13^. The resulting genetic mosaics reveal traits selected by host-vector-parasite interactions in a setting of ecological transition^12,13^. However, despite these insights, *P*. *knowlesi* isolates from both macaques and humans in Peninsular Malaysia are under-represented in analyses, and the genetic diversity in that geographical region is less clear.

One roadblock to large-scale genomic studies of clinical *P*. *knowlesi* parasites is that the majority of infections have a low parasitaemia, leading to samples with high levels of human compared to parasite DNA. Until now the WGS data for *Plasmodium* parasites has been obtained from venous blood of clinical cases that were filtered to remove human leukocytes, and therefore reduce human DNA “contamination”. However, this approach does not always yield sufficient parasite DNA for WGS. Recently, a selective whole genome amplification (SWGA) strategy has been used to sequence *P*. *falciparum* and *P*. *vivax* genomes from non-filtered blood and from dried blood spots of clinical samples^26–28^. The SWGA method uses oligonucleotides that preferentially bind with high frequency to the target DNA, but less frequently to the “contaminating” genome^29^. The high fidelity Phi29 polymerase is then used to amplify long segments of DNA. Here, we developed a SWGA approach for *P*. *knowlesi*, and sequenced 26 isolates across Malaysia, including from Peninsular and Borneo, revealing new insights into the population structure and evolution of this parasite.

## Results

### Selective whole genome amplification of *P*. *knowlesi* parasite DNA from clinical samples

We performed SWGA on *P*. *knowlesi* DNA samples obtained directly from human blood using six selected primers that specifically amplify the parasite genome and bind less frequently to the human genome (See Methods). The primer set had a mean binding frequency of at least once every 4,826 bp to the *P*. *knowlesi* genome, much higher than the once every 40,307 bp to the human genome. Binding sites that are sufficiently near each other, as obtained with the primer set for *P*. *knowlesi*, enable the branching and displacement actions of the Phi29 polymerase and increase the success of the genome amplification^30^.

**Figure 1:**
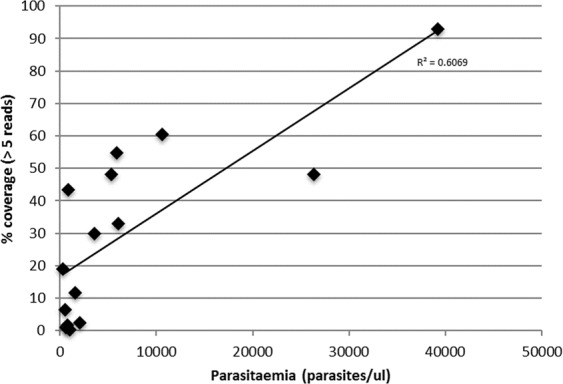
Correlation of parasitaemia (%) and genome coverage (>5 reads) in amplified DNA samples. Parasitaemia data was available for 13 amplified samples used in this study. An increase in coverage is observed with samples with a higher parasitaemia (R-squared = 0.6).

For ten samples, we performed WGS on both the non-amplified and the SWGA DNA. Both sets of samples (with and without amplification) were sequenced at the same theoretical depth, and we observed a significant increase (mean: 7.7-fold greater) in the proportion of reads that mapped to the *P*. *knowlesi* A1-H.1 reference genome after DNA amplification (Table 1, showing non-pooled sequencing results). As a result, amplified samples have higher genome coverage (mean: 6.8-fold greater) and a much greater number of callable SNPs (mean: 182-fold greater, with an average of 14,078 SNPs for no SWGA vs. 115,995 SNPs for SWGA) (Table 1). After amplification, higher coverage was observed in genes and intergenic regions (% of positions with a coverage >5-fold; within genes: no SWGA 5.3% vs. SWGA 43.3%; intergenic regions: no SWGA 4.1% vs. SWGA 30.4%) (Table 1). DNA from a further sixteen clinical isolates underwent SWGA and WGS. A trend towards improved coverage in samples with higher parasitaemia was observed (*R*^2^ = 0.6, Fig. 1), with superior results for the samples with ≥5,000 parasites/µl, consistent with data from *P*. *vivax* and *P*. *falciparum* isolates^27,28^. For the samples with <5,000 parasites/µl, results are more variable and do not correlate with an increase in parasitaemia. For these low-parasitaemia samples, the percentage of the genome coverage in excess of 5-fold ranged from 6% to 43% after amplification, and represent an average increase of 78% in coverage compared to non-amplified samples. This coverage difference led to an average of 66,143 callable SNPs post-amplification and 2,908 SNPs for non-amplified samples (Table 1). The average distribution of the genome coverage for the twenty high quality isolates undergoing SWGA, after applying quality filtering, is shown in Supplementary Fig. 1; a relatively uneven distribution of the coverage can be observed, which is similar to previous studies in other *Plasmodium* species^27,28^, but with no specific bias towards genic regions (Table 1). For samples with lower parasite densities, increased sequencing and merging of the resulting reads can lead to improved genome coverage, as shown for *P*. *vivax*^28^. Evidence of mixed infections (multiclonality) was detected in t*wo* SWGA samples, demonstrating that the method can amplify more than one clone present in an infection, as was observed for *P*. *vivax* amplified samples^28^.

**Table 1:** Comparison of whole genome sequencing before and after parasite enrichment using SWGA.

Sample ID	Parasitemia p/µl* (%)	Sample type	Reads aligned to *P*. *knowlesi* reference (%)*	% genome with coverage >5-fold	% genes with coverage >5-fold	% intergenic regions with coverage >5-fold	Mean coverage	Total N SNPs
1	320 (0.006%)	No SWGA	2.45	0.64	0.80	0.52	1.71	1,797
		SWGA	12.11	19.08	23.36	16.51	11.94	59,031
2	539 (0.01%)	No SWGA	0.81	0.04	0.03	0.04	1.28	15
		SWGA	3.66	6.49	7.54	5.97	4.38	14,746
3	851 (0.017%)	No SWGA	3.15	3.88	4.69	3.34	2.25	11,974
		SWGA	27.95	43.32	53.40	37.14	17.52	143,483
4	1581 (0.03%)	No SWGA	1.14	0.08	0.08	0.09	1.34	127
		SWGA	20.37	11.79	14.59	10.05	8.96	34,314
5	3554 (0.07%)	No SWGA	1.22	0.32	0.35	0.30	1.58	628
		SWGA	6.31	28.01	35.21	23.32	6.51	79,139
6	5300 (0.1%)	No SWGA	2.31	3.38	4.34	2.66	2.18	10,479
		SWGA	17.10	48.26	59.66	41.19	15.71	159,652
7	5875 (0.11%)	No SWGA	1.87	0.28	0.31	0.26	1.55	609
		SWGA	20.26	54.74	66.95	47.41	18.72	179,304
8	10634 (0.2%)	No SWGA	2.34	2.67	3.55	2.00	2.10	8,147
		SWGA	22.33	60.50	73.96	52.40	23.61	208,202
9	ND	No SWGA	10.59	2.51	2.58	2.56	2.14	2,197
		SWGA	44.54	32.03	38.01	28.87	8.97	119,157
10	26368 (0.5%)	No SWGA	11.01	31.36	36.22	28.77	4.05	104,805
		SWGA	42.21	48.26	59.89	40.99	18.04	162,920

*These results are from single runs, and not pooled samples (average of a total of 2 billion bp sequenced per sample (human and parasite)).

### *P*. *knowlesi* genetic variation and clustering of isolates

**Figure 2:**
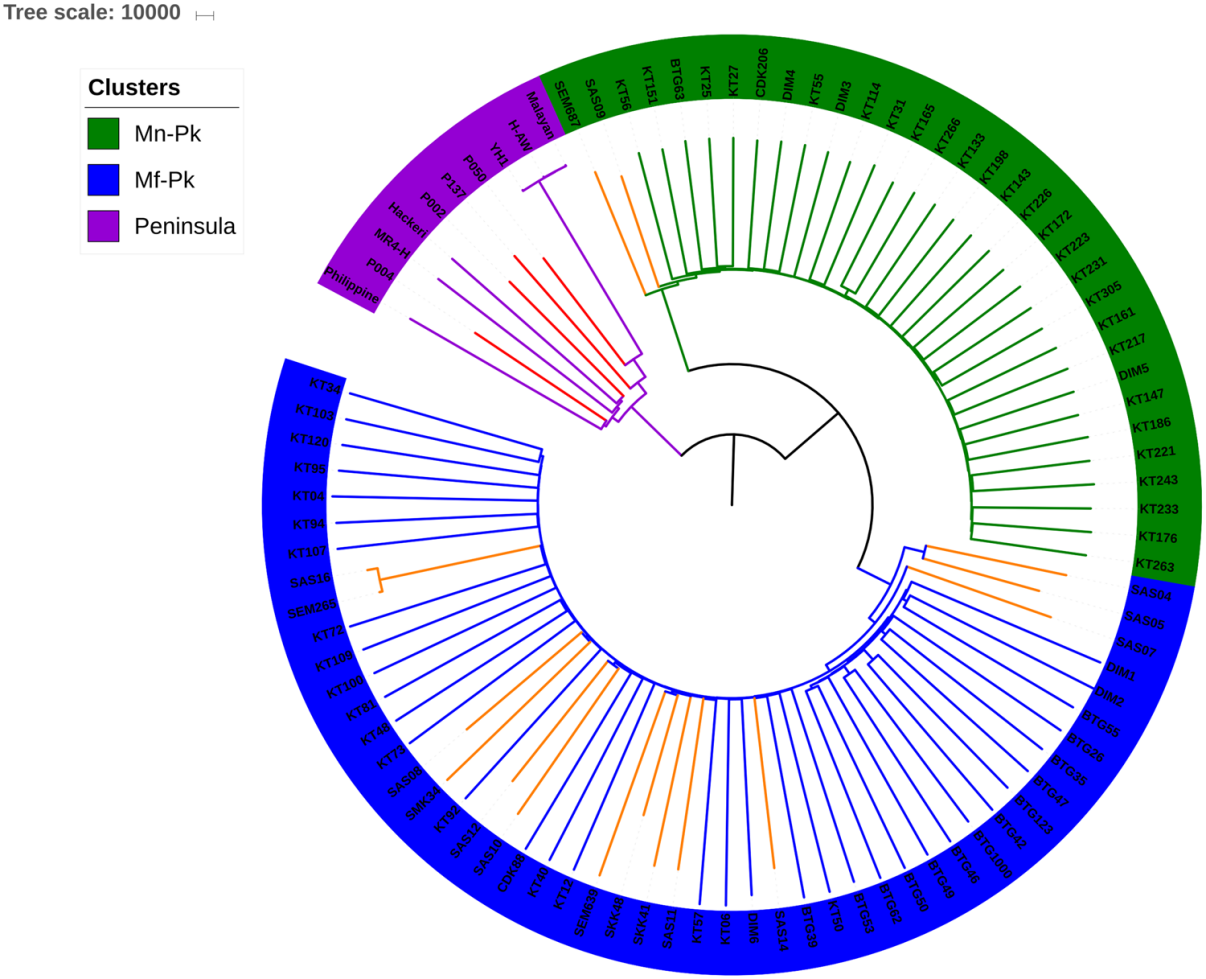
Neighbour-Joining tree for 103 *P*. *knowlesi* isolates shows three main clusters. The tree shows the expected split into three different clusters associated with: (i) Peninsular Malaysia (purple in tips), (ii) Malaysian Borneo *Macaca nemestrina* (*Mn-Pk*, green) and (iii) Malaysian Borneo *M*. *fascicularis* (*Mf-Pk*, blue). The tree also shows the correct positioning of the 4 newly generated Peninsular isolates (in red) within the Peninsular cluster, and the clustering of the 16 new Malaysian Borneo isolates from Sabah (in orange) within either of the *Mf-Pk or Mn-Pk* associated clusters. Bootstrapping was performed (n = 100) and all the nodes that split the relevant subpopulations presented with a value greater than 90.

The sequence data for the 26 new *P*. *knowlesi* isolates and 156 previously sequenced samples (see Methods) were mapped to the A1-H.1 reference genome^31^. The new genomes include recently collected Peninsular Malaysian isolates (n = 5) and clinical isolates from Sabah, Malaysia Borneo (n = 21). From the resulting alignments, 1,741,056 high quality SNPs were identified across the 14 chromosomes. Isolates with high levels of multiplicity of infection (MOI) (>15% of genome with MOI > 1 or >0.0004% of SNPs with mixed calls, see Supplementary Fig. 2) were excluded. In addition, isolates with overall low genome coverage (<30%) were excluded. As a result of these filters, 103 isolates, including 20 of the 26 isolates undergoing SWGA, were carried forward for further analysis (see Supplementary Table 1). A neighbour-joining tree was constructed using the SNP data (Fig. 2) and revealed 3 predominant clusters, consistent with recent findings^23,25^. In particular, these clusters relate to the specific geographic Peninsular-Malaysia subpopulation (purple, π = 3.4 × 10^−9^), and Borneo macaque *Mn-Pk* (green, π = 2.23 × 10^−9^) and *Mf-Pk* (blue, π = 3.29 × 10^−9^) associated subpopulations. The genetic distances between the populations, estimated as the average F_ST_^32^ for SNPs with minor allele frequencies >0.05, were 0.14 (*Mf-Pk* vs. *Mn-Pk*), 0.20 (*Mf-Pk* vs. Peninsular) and 0.28 (*Mn-Pk* vs. Peninsular). Furthermore, the tree showed a consistent positioning for SWGA isolates: 4 SWGA Peninsular isolates (red branches) clustered within the Peninsular Malaysia clade (purple), and of the 16 SWGA Sabah isolates, 2 and 14 clustered within the *Mn-Pk* (green) and *Mf-Pk* (blue) Borneo clades, respectively. This result demonstrates that the SWGA method can amplify all known sub-populations of *P*. *knowlesi*.

### Genetic exchange events in *P*. *knowlesi* isolates from Peninsular Malaysia

**Figure 3:**
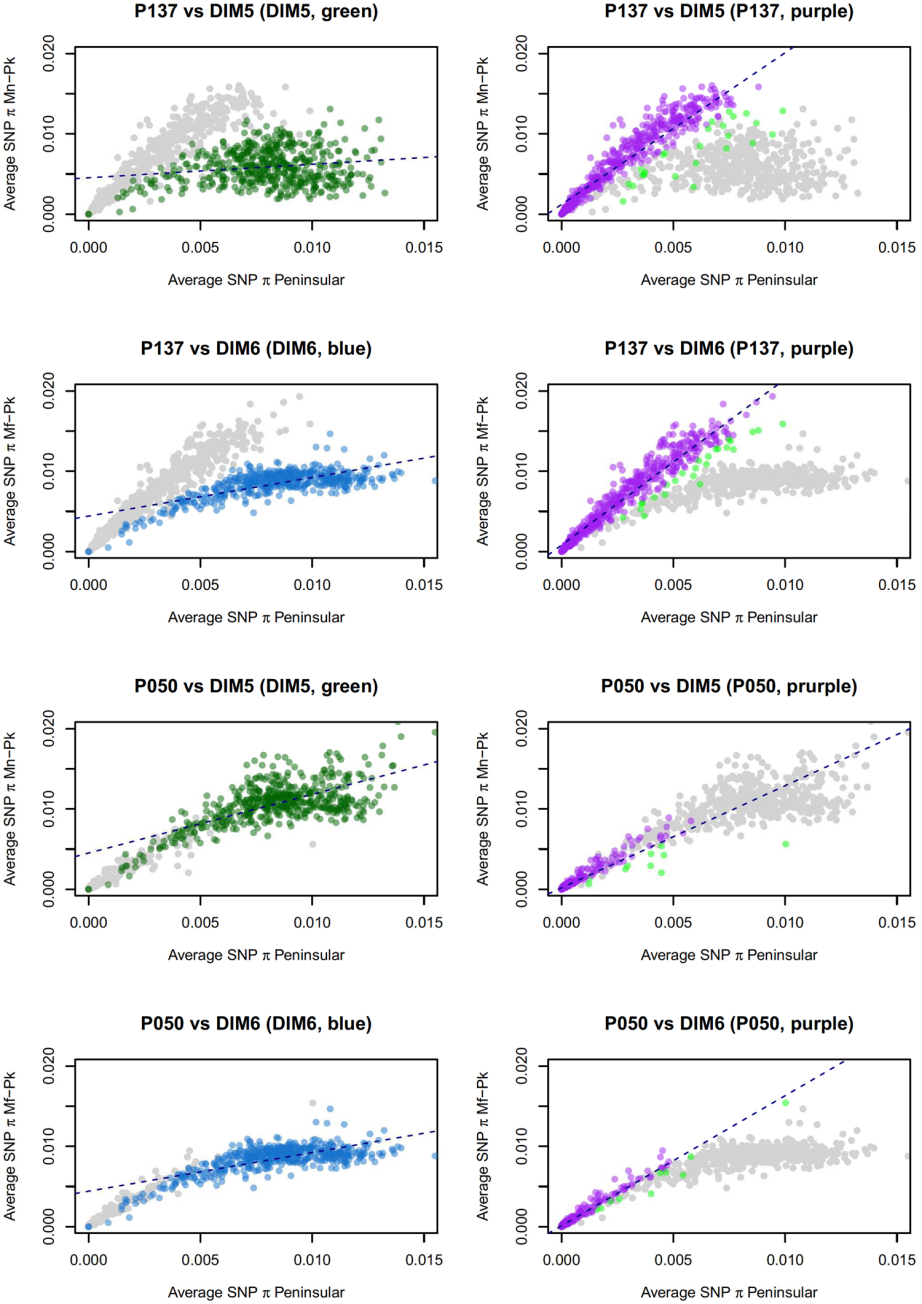
*P*. *knowlesi* isolates with the highest genomic coverage from Peninsular Malaysia (P137 and P050) present with genetic exchange events from the *Mn-Pk* sub-population. Peninsular isolate P137 was compared to DIM5 (top two panels) as a representative of the *Mn-Pk* cluster, and DIM6 (second row panels) of the *Mf-Pk* cluster; these isolates were selected based on sequencing quality and completeness, and an absence of evidence of multiplicity of infection. Isolate P050 was compared to the same isolates in the bottom 4 panels. On the top left panel each green dot represents a 50 kbp section of the DIM5 genome. Its position on the X-axis is defined by the average SNP *π* obtained by comparing its sequence in a pairwise manner to the same syntenic genome 50 kbp fragment in each isolate in the *Mn-Pk* cluster; in the Y-axis the average SNP π is compared to the same fragment of the Peninsular isolates. This average SNP π defines the similarity of each dot to the different clusters. The top most right panel represents the same data as the top left most panel with the P137 50 kbp fragments highlighted in purple for clarity. The same analysis was conducted in the second row of panels but using a *Mf-Pk* cluster isolate and the average SNP *π* to *Mf-Pk* as the X-axis. The dashed line represents the linear regression for the coloured dots in each plot, and the regions of interest were identified (in light green in the right panels) by finding the fragments of the Peninsular isolate genomes that presented low similarity to the Peninsular cluster and high similarity to either the *Mn-Pk* (green) or *Mf-Pk* (blue). This approach accounts for the highest residuals. After further filtering through the assessment of individual genes, we report the set of results in Supplementary Table 2.

It has been shown that the subpopulations of *P*. *knowlesi* in Malaysian Borneo, although presenting a strong genetic differentiation, are not genetically isolated. In particular, we have identified genetic exchanges predominantly between the *Mf-Pk* and *Mn-Pk* clusters^23^. We sought to investigate whether these events are also found in the clinical isolates from Peninsular Malaysia, by estimating SNP nucleotide diversity (*SNP π*) across the genome in sliding 50 kbp windows. In the two isolates where genome coverage was highest (P137 and P050), we identified several regions with an exceptional increase in similarity with the *Mn-Pk* cluster and reduced similarity with the Peninsular Malaysia cluster (Fig. 3). Analysis of the haplotypes for each individual isolate confirmed exchange events. The analysis of the individual sequence haplotypes of genes in the identified regions showed mis-clustering in a neighbour-joining tree when compared to the whole genome clustering patterns. These genes are represented in Supplementary Table 2. All the events observed were associated with genetic exchanges from *Mn-Pk* cluster haplotypes into the Peninsular Malaysia genomes and spanned mostly subtelomeric regions in chromosomes 1, 2, 7, 9, 10, 11, 12, 13 and 14. A high proportion of *Plasmodium* exported proteins with unknown function were found to be affected by the exchange, as well as tryptophan-rich antigens and lysophospholipases, genes associated with parasite invasion (*Normocyte Binding Protein Xa* (NBPXa), *Duffy Binding Protein beta* (*DBPβ*))^33^, and a cytoadherence linked asexual protein gene (*PKNH_1401300*). These results could indicate that the exchange events found in Peninsular Malaysia may affect vertebrate host-related factors in the erythrocytic stages of the parasite life cycle and could potentially impact the invasion of human red blood cells by *P*. *knowlesi*.

**Figure 4:**
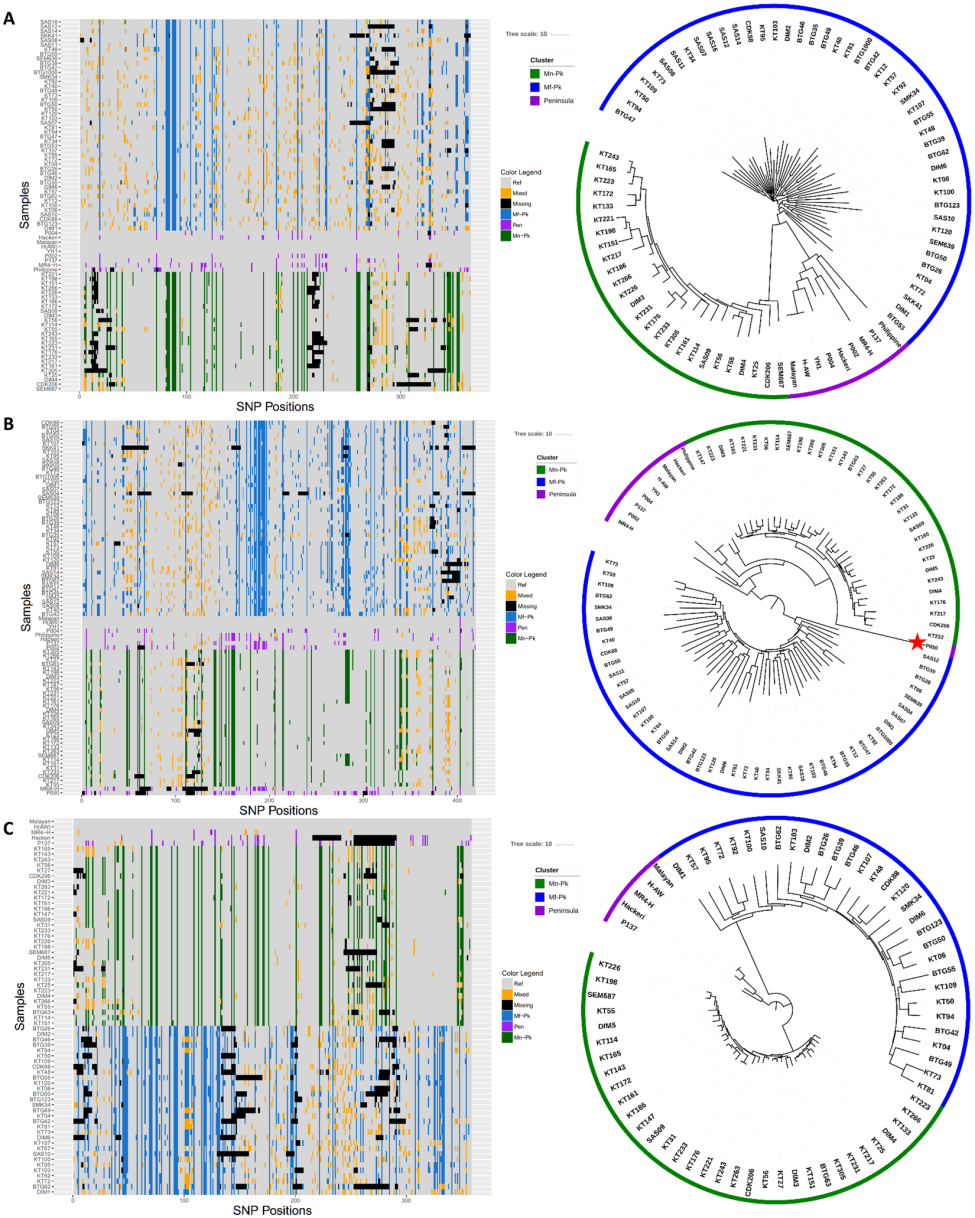
Haplotype plot and neighbour-joining tree for three Duffy Binding Protein invasion genes ((**A**) *DBPα*, (**B**) *DBPβ*, (**C**) *DBPγ*) provides insights into the population dynamics of the different haplotypes. Only isolates with at least 30-fold coverage across the gene were used in each plot: 81 isolates for *DBPα*, 89 isolates for *DBPβ* and 70 isolates for *DBPγ*. A strong genetic divergence of the sequences from the different clusters was found for each of the 3 genes, and the Peninsular cluster had the highest diversity across all 3 genes (**A**–**C**). Red stars indicate isolates with differences in subpopulation clustering when compared to whole genome clustering, suggesting a genetic exchange. Bootstrapping was performed (n = 100) and all the nodes that split the relevant subpopulations and/or exchange events presented with a value greater than 84.

**Figure 5:**
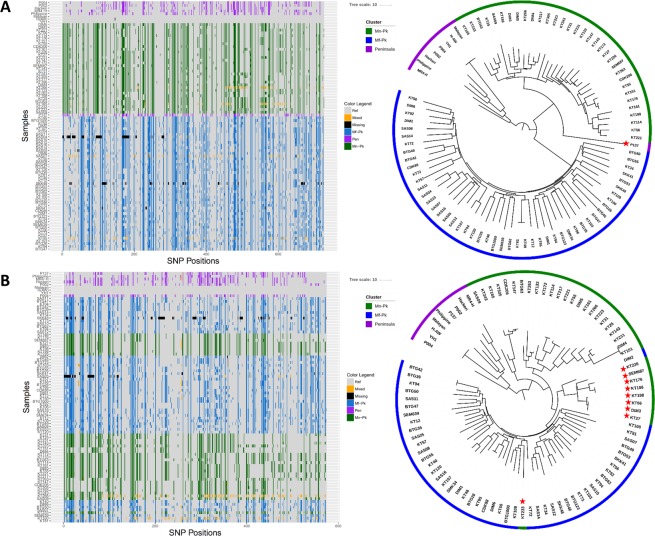
Haplotype plots and neighbour-joining trees for two Normocyte Binding Protein invasion genes ((**A**) *NBPXa*, (**B**) *NBPXb*) provides insights into the population dynamics of the gene haplotypes. Only isolates with at least 30-fold coverage across the gene were used in each plot: 88 isolates for *NBPXa* and 90 isolates for *NBPXb*. Red stars indicate isolates with differences in subpopulation clustering when compared to whole genome clustering, suggesting a genetic exchange. For the *NBPXa* gene (**A**), evidence of strong genetic divergence of the sequences from the different clusters was found. However, the *NBPXb* gene (**B**, right) presented a fairly distinct pattern of diversity. The clusters have small genetic distances between themselves, making the separation between them less obvious. Bootstrapping was performed (n = 100) and all the nodes that split the relevant subpopulations and/or exchange events presented with a value greater than 82.

Following the discovery of *P*. *knowlesi* invasion-related genes in the exchanged regions, we performed a comprehensive analysis of the genetic diversity harboured by the five reticulocyte binding like (*RBL*) and Duffy binding protein like (*DBP*) genes involved in erythrocyte invasion: *DBPα* (81 isolates, Fig. 4A), *DBPβ* (89 isolates, Fig. 4B), *DBPγ* (70 isolates, Fig. 4C), *NBPXa* (88 isolates, Fig. 5A), and *NBPXb* (90 isolates, Fig. 5B). For each of the high-quality isolates with coverage in excess of 30-fold across the different genes, we characterised their “invasion” haplotypes (Figs 4 and 5, left) and compared their resulting position on neighbour-joining trees to their expected clustering based on WGS data (Figs 4 and 5, right). For each of the 5 genes, there was evidence of strong genetic divergence of the sequences across the different clusters. Across all 5 genes, the Peninsular Malaysia cluster presented with marginally greater nucleotide diversity (Peninsular Malaysia: mean = 1.88 × 10^−5^; range = 1.63 × 10^−5^–2.17 × 10^−5^) when compared to the other two clusters (*Mf-Pk*: mean = 1.85 × 10^−5^, range = 1.15 × 10^−5^–2.71 × 10^−5^; *Mn-Pk*: mean = 1.01 × 10^−5^, range = 0.78 × 10^−5^–1.42 × 10^−5^).

For all the isolates, *DBPα* and *DBPγ* gene sequence-based clusters matched those from whole genome data. For *DBPβ*, the overall clustering pattern was still present, but one isolate from Peninsular Malaysia (P050; Fig. 4B, right; red star) had strong evidence of genetic exchanges with the *Mn-Pk* cluster. The longer branch length of the P050 isolate in the tree, where the genetic exchange is found, reveals a stronger genetic difference compared to the other *Mn-Pk* DBP*β* haplotypes, thereby indicating that this exchange event could be non-recent. Analysis of the neighbouring genes revealed that this exchange spanned from approximately 31 kb to 96 kb in chromosome 14, affecting 14 genes. This observation is confirmed by the partial similarity of the P050 haplotype with the *Mn-Pk* haplotypic patterns (Fig. 4B, left). There was also evidence of genetic exchange events in the *NBPXa*, which had the lowest overall genetic diversity of all RBL/DBP genes (mean = 1.20 × 10^−5^). For example, the Peninsular Malaysia isolate P137 appears to have incorporated a haplotype from the *Mn-Pk* cluster, and its partial similarity to the current *NBPXa* haplotypes of the *Mn-Pk*’s clade suggests it could also be a non-recent event (Fig. 5A, right). An analysis of neighbouring genes revealed that the exchange event spanned from approximately 3,157 kb to 3,204 kb in chromosome 14, affecting 8 genes. Finally, *NBPXb* gene had low diversity, with intra-cluster genetic distances being smaller than those found in the DBP genes. Several genetic exchange events were identified, where 9 out of 33 (27%) of the *Mn-Pk* cluster isolates presented with *Mf-Pk* type haplotypes (Fig. 5B, right; red stars). Most of these isolates with genetic exchange evidence clustered together and were separated from the *Mf-Pk* isolates, which could be a reflection of a unique old genetic exchange event. This observation is consistent with the *NBPXb* gene, and not its neighbouring loci, being exchanged. The isolate KT233 positioned in the “*Mn-Pk”* group using all SNPs, has a much similar *NBPXb* haplotype to those found in the *Mf-Pk* samples, reflecting a more recent exchange event.

## Discussion

The SWGA approach implemented led to reliable sequence data being generated for parasite isolates obtained from unprocessed human blood, and belonging to the three currently known subpopulations of *P*. *knowlesi*. This method is cost-effective, does not require sample processing at the time of collection, requires low quantities of input DNA, and is easy to implement. Importantly, the approach permits the genomic analysis of isolates that would otherwise be very difficult to investigate, as demonstrated by the poor WGS results of the non-SWGA DNA when compared with their respective SWGA samples. A neighbour-joining tree based on the SNP data generated revealed 3 predominant clusters, consistent with recent findings, and the positioning of the SWGA isolates confirmed their origin. The four recent Peninsular Malaysia isolates clustered closely with the long-term maintained samples originating from different regions in Peninsular Malaysia and the Philippines. Of the sixteen isolates originating from Sabah, two belonged to the *Mn-Pk* associated cluster, and fourteen belonged to the *Mf-Pk* associated cluster. This finding is consistent with the higher proportions of samples circulating in humans belonging to the *Mf-Pk* cluster^34^ and confirms the presence of both Borneo macaque host-related subpopulations in Sabah.

Previous population genetics studies on *P*. *knowlesi* subpopulations among human and macaque isolates from Malaysian Borneo provided evidence that chromosomal-segment exchanges between subpopulations have occurred recently^23^. This observation could be indicative of subpopulations that diverged in isolation and have re-connected, possibly due to deforestation and disruption of wild macaque habitats. Up until now these genetic exchanges had only been observed in parasites from Malaysian Borneo^10^, but the inclusion of recent Peninsular Malaysia isolates allowed us to scan for more widespread events. We identified regions that presented evidence of genetic exchange, in particular, with exceptionally high average SNP diversity when compared to the Peninsular isolates and exceptionally low diversity when compared to *Mn-Pk* or *Mf-Pk* isolates. This approach highlighted the presence of such events in two Peninsular samples, where the identified regions were found to be genetic exchanges with *Mn-Pk* type haplotypes and present across multiple chromosomes. This work shows that genetic exchange events are more widespread than previously thought, and they also affect the geographic subpopulation of Peninsular Malaysia. This finding is consistent with microsatellite analyses that have identified traces of Borneo-associated clusters in regions of Peninsular Malaysia^34,35^.

Regions identified with genetic exchange events were enriched with large multi-gene families coding for *Plasmodium* exported proteins and tryptophan-rich antigens, as well as loci associated with erythrocyte invasion by *P*. *knowlesi*. These findings contrast with our previous results found in isolates from Borneo^23^, where the genes involved in genetic exchange events were enriched by mosquito-stage related genes. This difference in gene ontology could suggest that there are different factors driving the exchanges between geographical regions. We found that all known RBL/DBP invasion genes (*DBP α*, *β* and *γ*; *NBP Xa* and *Xb*) are highly differentiated and cluster the isolates into three subpopulations. This clustering is consistent with vertebrate host-related factors being one of the main drivers for their genetic differentiation; although the Peninsular subpopulation is assumed to be a geographic subdivision rather than a macaque host-associated cluster. Across all five loci the *Mf-Pk* group is the most genetically diverse. The *DBPα* and *DBPγ* loci did not present with any genetic-exchange patterns. For the *NBPXb* gene, the three subpopulations were not as strongly differentiated as in other genes, and some of the *Mn-Pk* isolates (including from Sabah) presented with genetic exchanges with the *Mf-Pk* subpopulation. These genetic exchanges could be related to the adaptation of the parasites to different vertebrate hosts, especially as it has been shown that the two Borneo subpopulations can be found in both species of macaques^24,25,34^. Furthermore, the events observed here could involve yet another subpopulation of a Peninsular *Mn-Pk* type of *P*. *knowlesi*, especially as the level of sampling currently is very low and the haplotypes do show a degree of differentiation with the other *Mn-Pk* haplotypes. Other genes such as *DBPβ* and *NBPXa* presented genetic exchange events with *Mn-Pk* into the Peninsular subpopulation. *NBPXa* has the lowest level of genetic diversity, which suggests that this gene is highly conserved across subpopulations. This finding is important because *NBPXa* is required for the invasion of human red blood cells *in vitro*^33^. It has been shown that different haplotypes in the *DBPα* region II have differential binding affinities to the DARC receptor in human erythrocytes^36^. Therefore, these genetic exchanges affecting genes involved in invasion may reflect an adaptation to a new vertebrate host and may confer improved binding and increase invasion efficiency. It will be important to investigate whether changes in the haplotypes of other genes involved in erythrocyte invasion affect the ability of the parasite to invade and multiply in human cells. Genetic interactions between invasion genes may assist parasites with adapting more efficiently to humans and facilitate transmission, which could hamper malaria elimination efforts.

Overall, by establishing an effective SWGA strategy for *P*. *knowlesi*, it will be possible to perform much needed large-scale WGS studies of the parasite genomic diversity across Asia, as well as investigate important fundamental biology, such as the genetics underlying mechanisms for erythrocyte invasion.

## Methods

### Sample collection and preparation

For this project we use *P*. *knowlesi* DNA samples from Sabah in Malaysian Borneo (n = 21) (provided by the Menzies School of Health Research) and from Peninsular Malaysia (n = 5) (provided by the University of Malaya). Samples from Sabah were obtained from patients enrolled as part of clinical malaria studies conducted from 2010 to 2014^10,37^. Ethical approval for these studies was obtained from the Ministry of Health, Malaysia, and Menzies School of Health Research, Australia. Samples from Peninsular Malaysia were collected from patients admitted to University Malaya Medical Centre (UMMC), Kuala Lumpur, from July 2008 to December 2014^38^. Ethical approval was provided by the University of Malaya Medical Centre Medical Ethics Committee (MEC Ref. No: 817.18). Informed consent was obtained for study participation in both Sabah and Kuala Lumpur sites. All methods were performed in accordance with the relevant guidelines and regulations in both Sabah and Kuala Lumpur sites. All DNA samples were quantified using the Qubit Fluorometer using the dsDNA high sensitivity method (Invitrogen). All samples were screened by PCR targeting the genes encoding the *Plasmodium* 18S rRNA^39^. Confirmation of *P*. *knowlesi* mono-infection was performed using a heminested PCR assay based on a *P*. *knowlesi* specific conserved *SICAvar* region, to overcome possible cross reactivity between *P*. *knowlesi* and other *Plasmodium* species^40,41^. The relative amounts of parasite and human DNA in each sample was determined using a qPCR protocol using primers and probes specific for each species^42–44^. Pure human and *P*. *knowlesi* standard controls (range 0.0001–100 ng/ul concentrations) were included to determine the relative concentration (ng/ul) of each organism’s DNA in a sample.

### Primer design for selective whole genome amplification

The *swga* program (www.github.com/eclarke/swga) was used to identify primer sets that preferentially amplify the *P*. *knowlesi* genome, providing as input the new A1-H.1 reference for the *P*. *knowlesi* human-adapted line A1-H.1^31^ and the established human reference human_g1k_v37 (ftp://ftp.1000genomes.ebi.ac.uk). The resulting ten best sets consisted of combinations of four to six oligonucleotides each, with several overlapping primers, including two that were present in all sets. The set with the lowest Gini index and perfectly even binding across the genome consists of the following six primers: 5′-ATAATC*G*T-3′, 5′-ATTATC*G*T-3′, 5′-CGAAAT*A*G-3′, 5′-CGATAA*A*G-3′, 5′-GAATAA*C*G-3′ and 5′-TCGTAA*T*A-3′; where asterisks represent phosphorothioate bonds to prevent primer degradation by the exonuclease activity of the Phi29 polymerase

### Selective whole genome amplification

Selective whole genome amplification (SWGA) was performed according to published protocols^27^. All SWGA reactions were carried out in the UV Cabinet for PCR Operations (UV-B-AR, Grant-Bio) to minimize contamination. SWGA reactions were performed containing a maximum of 50 ng of total input genomic DNA (and a minimum of 5 ng), 5 µl of 10 x Phi29 DNA Polymerase Reaction Buffer (New England BioLabs), 0.5 µl of Purified 100x BSA (New England BioLabs), 0.5 µl of 250 µM Primer mix of Pkset1, 5 µl 10 mM dNTP (Roche), 30 units Phi29 DNA Polymerase (New England BioLabs) and Nuclease-Free Water (Ambion, The RNA Company) to reach a final reaction volume of 50 µl. The reaction was carried out on a thermocycler with the following step-down program: 5 minutes at 35 °C, 10 minutes at 34 °C, 15 minutes at 33 °C, 20 minutes at 32 °C, 25 min 31 °C, 16 hours at 30 °C and 10 minutes at 65 °C. The SWGA samples were diluted 1:1 with EB buffer (Qiagen) and the reaction was purified using the AMPure XP beads (Beckman-Coulter), using a sample to beads ratio of 1:1 according to the protocol.

### Whole-genome sequencing, bioinformatics analysis and population genetics

The SWGA products and unamplified DNA were sequenced on an Illumina MiSeq or HiSeq4000 platform. DNA and SWGA Libraries for MiSeq were prepared using the QIAseq FX DNA Library Kit (Qiagen) as per manufacturer’s instructions. A twenty-minute fragmentation step was optimized for *Plasmodium* samples. For the HiSeq runs, libraries were prepared using the NEB Next Ultra DNA Library Prep Kit for Illumina (from New England BioLabs Inc., E7370). All samples were run using 150 bp paired-end reads. The raw sequence data for the isolates was aligned against the new reference for the human-adapted line A1-H.1 (no regions were excluded for analysis)^31^ using *bwa-mem* software with default settings^45^. In order to establish the amount of human DNA in the isolate data, the sequence data was mapped to the GRCh37 human reference genome (NCBI; latest version, as accessed on 23/11/2018) using *bwa-mem* software with default parameters. WGS data from an extra 156 publicly available samples were also used for analysis (sourced from^24,25,46^, where sequencing accession numbers are listed). SNPs were called using the *Samtools* software suite^47^, and those of high quality were retained using previously described methods (phred score > 30, 1 error per 1 kbp)^23^. Samples with high levels of multiplicity of infection were detected using estMOI software^48^. For comparisons between populations, we applied principal component analysis (using *R* core functions *dist* and *cmd*.*scale;* results not presented) and neighbour-joining tree (using *ape* package in R^49^) approaches. These clustering approaches were implemented on a *Manhattan* distance matrix of pairwise identity by state values calculated from the SNP data. Nucleotide diversity (π) for pairwise isolate comparisons was calculated using in-house R scripts adapted from a previous study^50^. These scripts resulted in the number of SNP differences between the two sequences divided by the length of the DNA fragment. For population comparisons the function *nuc*.*div* from the *pegas* package in *R* was used^51^. *R-base* scripts were used to perform linear regression analyses for the *SNP π* plots. For the construction of the robust haplotypes of the invasion-related genes (*DBPα*, *DBPβ*, *DBPγ*, *NBPXa* and *NBPXb*) we used only reads mapped with high quality and discarded any SNPs with high levels of missing alleles or evidence of multiplicity of infection (MOI > 1). Genetic differentiation between subpopulations was calculated using the average F_ST_^32^ value for each pairwise comparison, using only SNPs with minor allele frequencies in excess of 5%.

## Supplementary information

Supplementary figures and tables

## Data Availability

Previously published data can be found on the ENA using the Run accession codes in Supplementary Table 1. The newly generated data can be found in the ENA study accession number ERP110368.
