# Supplementary material for: Whole genome sequencing of amplified Plasmodium knowlesi DNA from unprocessed blood reveals genetic exchange events between Malaysian Peninsular and Borneo subpopulations
Source: Sci Rep. 2019 Jul 8;9:9873. doi: 10.1038/s41598-019-46398-z (PMC6614422; doi:10.1038/s41598-019-46398-z)
Supplement: Supplementary file 1 — Supplementary figures and tables [file 41598_2019_46398_MOESM1_ESM.pdf]

Whole genome sequencing of amplified *Plasmodium knowlesi* DNA from unprocessed blood reveals genetic exchange events between Malaysian Peninsular and Borneo subpopulations

Ernest Diez Benavente

Ana Rita Gomes

Jeremy Ryan De Silva

Matthew Grigg

Harriet Walker

Bridget E. Barber

Timothy William

Tsin Wen Yeo

Paola Florez de Sessions

Abhinay Ramaprasad

Amy Ibrahim

James Charleston

Martin L. Hibberd

Arnab Pain

Robert W. Moon

Sarah Auburn

Lau Yee Ling

Nicholas M. Anstey

Taane G. Clark

Susana Campino

Supplementary table 1

The samples analysed

Sample	Code	Area	estMOI*	Group**
ERR274221	DIM1	Sarikei	0.8	*Mf-Pk*
ERR274222	DIM2	Sarikei	0.7	*Mf-Pk*
ERR274224	DIM3	Sarikei	0.6	*Mn-Pk*
ERR274225	DIM4	Sarikei	0.5	*Mn-Pk*
ERR366425	DIM5	Sarikei	0.3	*Mn-Pk*
ERR366426	DIM6	Sarikei	0.2	*Mf-Pk*
ERR985372	BTG1000	Betong	0.8	*Mf-Pk*
ERR985373	BTG123	Betong	0.7	*Mf-Pk*
ERR985374	BTG26	Betong	1.6	*Mf-Pk*
ERR985375	BTG35	Betong	6.8	*Mf-Pk*
ERR985376	BTG39	Betong	9.9	*Mf-Pk*
ERR985377	BTG42	Betong	1.0	*Mf-Pk*
ERR985378	BTG46	Betong	1.5	*Mf-Pk*
ERR985379	BTG47	Betong	1.2	*Mf-Pk*
ERR985380	BTG49	Betong	5.9	*Mf-Pk*
ERR985381	BTG50	Betong	1.4	*Mf-Pk*
ERR985382	BTG53	Betong	1.3	*Mf-Pk*
ERR985383	BTG55	Betong	3.6	*Mf-Pk*
ERR985384	BTG62	Betong	3.0	*Mf-Pk*
ERR985385	CDK88	Kapit	1.1	*Mf-Pk*
ERR985386	KT03	Kapit	22.8	*Mf-Pk*
ERR985387	KT04	Kapit	2.7	*Mf-Pk*
ERR985388	KT06	Kapit	1.0	*Mf-Pk*
ERR985389	KT100	Kapit	2.8	*Mf-Pk*
ERR985390	KT103	Kapit	0.5	*Mf-Pk*
ERR985391	KT107	Kapit	0.6	*Mf-Pk*
ERR985392	KT109	Kapit	0.6	*Mf-Pk*
ERR985393	KT120	Kapit	0.5	*Mf-Pk*
ERR985394	KT12	Kapit	1.0	*Mf-Pk*
ERR985395	KT26	Kapit	50.4	*Mf-Pk*
ERR985396	KT29	Kapit	21.0	*Mf-Pk*
ERR985397	KT30	Kapit	22.3	*Mf-Pk*
ERR985398	KT34	Kapit	1.2	*Mf-Pk*
ERR985399	KT40	Kapit	1.3	*Mf-Pk*
ERR985400	KT48	Kapit	1.0	*Mf-Pk*
ERR985401	KT50	Kapit	1.0	*Mf-Pk*
ERR985402	KT57	Kapit	0.7	*Mf-Pk*
ERR985403	KT72	Kapit	0.6	*Mf-Pk*
ERR985404	KT73	Kapit	1.2	*Mf-Pk*
ERR985405	KT77	Kapit	30.9	*Mf-Pk*
ERR985406	KT81	Kapit	0.5	*Mf-Pk*
ERR985407	KT92	Kapit	0.8	*Mf-Pk*
ERR985408	KT94	Kapit	0.6	*Mf-Pk*
ERR985409	KT95	Kapit	3.5	*Mf-Pk*
ERR985410	BTG44	Betong	32.7	*Mn-Pk*
ERR985411	BTG63	Betong	0.8	*Mn-Pk*

ERR985412	CDK206	Kapit	0.8	*Mn-Pk*
ERR985413	KT114	Kapit	0.5	*Mn-Pk*
ERR985414	KT25	Kapit	0.8	*Mn-Pk*
ERR985415	KT27	Kapit	0.9	*Mn-Pk*
ERR985416	KT31	Kapit	1.2	*Mn-Pk*
ERR985417	KT42	Kapit	29.9	*Mn-Pk*
ERR985418	KT55	Kapit	0.7	*Mn-Pk*
ERR985419	KT56	Kapit	4.5	*Mn-Pk*
PKAP_KK41	SKK41	Sabah	0.3	*Mf-Pk*
PKAP_KK48	SKK48	Sabah	3.0	*Mf-Pk*
PKAP_MK34	SMK34	Sabah	0.8	*Mf-Pk*
PKAP_QEM265	SEM265	Sabah	0.0	*Mf-Pk*
PKAP_QEM639	SEM639	Sabah	9.0	*Mf-Pk*
PKAP_QEM687	SEM687	Sabah	0.6	*Mn-Pk*
PKAS04_com	SAS04	Sabah	0.0	*Mf-Pk*
PKAS05_com	SAS05	Sabah	1.8	*Mf-Pk*
PKAS07_com	SAS07	Sabah	7.5	*Mf-Pk*
PKAS08_com	SAS08	Sabah	0.3	*Mf-Pk*
PKAS09_com	SAS09	Sabah	0.5	*Mn-Pk*
PKAS10_com	SAS10	Sabah	0.6	*Mf-Pk*
PKAS11_com	SAS11	Sabah	0.5	*Mf-Pk*
PKAS12_com	SAS12	Sabah	0.3	*Mf-Pk*
PKAS13_com	SAS13	Sabah	18.0	*Mf-Pk*
PKAS14_com	SAS14	Sabah	0.5	*Mf-Pk*
PKAS15_com	SAS15	Sabah	20.9	*Mf-Pk*
PKAS16_com	SAS16	Sabah	0.5	*Mf-Pk*
SRR2221468	Hackeri	Clinic	1.2	Peninsular
SRR2222335	H(AW)	Clinic	0.0	Peninsular
SRR2225467	Malayan	Clinic	10.7	Peninsular
SRR2225571	MR4-H	Clinic	0.6	Peninsular
SRR2225573	Philippine	Clinic	0.8	Peninsular
SRR3135172	YH1	Clinic	5.6	Peninsular
swga002d	P002	Peninsular	0.8	Peninsular
swga004	P004	Peninsular	3.2	Peninsular
ERR2214837	KT133	Kapit	0.3	*Mn-Pk*
ERR2214838	KT143	Kapit	0.2	*Mn-Pk*
ERR2214839	KT147	Kapit	0.3	*Mn-Pk*
ERR2214840	KT151	Kapit	0.3	*Mn-Pk*
ERR2214841	KT161	Kapit	0.3	*Mn-Pk*
ERR2214842	KT165	Kapit	14.6	*Mn-Pk*
ERR2214843	KT172	Kapit	0.2	*Mn-Pk*
ERR2214844	KT176	Kapit	0.3	*Mn-Pk*
ERR2214845	KT186	Kapit	1.0	*Mn-Pk*
ERR2214846	KT198	Kapit	0.3	*Mn-Pk*
ERR2214847	KT217	Kapit	0.4	*Mn-Pk*
ERR2214848	KT221	Kapit	0.2	*Mn-Pk*
ERR2214849	KT223	Kapit	0.3	*Mn-Pk*
ERR2214850	KT224	Kapit	19.0	*Mn-Pk*
ERR2214851	KT226	Kapit	0.6	*Mn-Pk*
ERR2214852	KT231	Kapit	0.2	*Mn-Pk*
ERR2214853	KT233	Kapit	0.2	*Mn-Pk*

ERR2214854	KT243	Kapit	0.4	*Mn-Pk*
ERR2214855	KT263	Kapit	0.4	*Mn-Pk*
ERR2214856	KT266	Kapit	13.6	*Mn-Pk*
ERR2214857	KT305	Kapit	0.7	*Mn-Pk*
swga_0137_f	P137	Peninsular	0.4	Peninsular
swga_050_f	P050	Peninsular	0.1	Peninsular

***** Percentage of the genome that shows evidence of multiplicity of infection (MOI) > 1 based on estMOI software ***

** Borneo Malaysia (*M. nemestrina* (*Mn-Pk*) and *M. fascicularis* (*Mf-Pk*) macaques and humans)

******* Assefa, S. A. *et al.* estMOI: estimating multiplicity of infection using parasite deep sequencing data. *Bioinformatics* **30**, 1292–1294 (2014).

Supplementary table 2

Genetic regions with evidence of genetic exchange* for the P050 and P137 samples

Chr.	Start	End	Samples	Genes affected
1	1	50000	P050, P137	*PKNH_0100500 (hypothetical protein, conserved), PKNH_0100600 (CPPUF**), PKNH_0100700 (CPPUF)*
2	750000	787646	P050	*PKNH_0211700 (CPPUF), PKNH_0211800 (NFS), PKNH_0211900 (DNMT)*
7	1	50000	P050	*PKNH_0700700 (CPPUF), PKNH_0700800 (RER1), PKNH_0700900 (RAB7)*
7	1250000	1300000	P137	*PKNH_0728200 (CPPUF), PKNH_0728300 (CPPUF), PKNH_0728400 (CPPUF), PKNH_0728800 (MSP1P), PKNH_0728900 (MSP1), PKNH_0729000 (CPPUF), PKNH_0729100 (diacylglycerol kinase, putative), PKNH_0729200 (CYP72), PKNH_0729300 (CPPUF)*
7	1450000	1500000	P050, P137	*PKNH_0734500 (PEPUF***), PKNH_0734600 (CPPUF), PKNH_0734700 (ETRAMP), PKNH_0734800 (Plasmodium exported protei, PHIST), PKNH_0734900 (PEPUF), PKNH_0735000 (lysophospholipase, putative)*
9	1	50000	P137	*PKNH_0900100 (hypothetical protein), PKNH_0900200 (PEPUF), PKNH_0900300 (hypothetical protein)*
9	50000	100000	P050, P137	*PKNH_0900400 (hypothetical protein), PKNH_0900500 (PEPUF), PKNH_0900600 (CPPUF)*
10	1450000	1500000	P137	*PKNH_1032400 (SBP1), PKNH_1032500 (tryptophan-rich antigen, putative), PKNH_1032600 (PEPUF), PKNH_1032800 (PEPUF)*
11	2300000	2350000	P050, P137	*PKNH_1149000 (CPPUF), PKNH_1149100 (PEPUF), PKNH_1149200 (PEPUF), PKNH_1149400 (PEPUF), PKNH_1149600 (hypothetical protein)*
12	2050000	2100000	P050, P137	*PKNH_1246200 (CPPUF), PKNH_1246500 (PEPUF), PKNH_1246600 (PEPUF)*
12	2100000	2150000	P050, P137	*PKNH_1246800 (PEPUF), PKNH_1246900 (PEPUF), PKNH_1247000 (PEPUF)*
12	2150000	2200000	P050	*PKNH_1247800 (PEPUF), PKNH_1247900 (CPPUF)*
13	1	50000	P050, P137	*PKNH_1300400 (Plasmodium exported protein, PHIST), PKNH_1300500 (tryptophan-rich antigen, putative), PKNH_1300600 (tryptophan-rich antigen, putative), PKNH_1300700 (tryptophan/threonine-rich antigen, putative), PKNH_1300900 (PEPUF)*
13	50000	100000	P050, P137	*PKNH_1301000 (tryptophan-rich antigen, putative), PKNH_1301100 (lysophospholipase, putative), PKNH_1301300 (PPUF), PKNH_1301800 (CPPUF), PKNH_1302100 (mitochondrial phosphate carrier protein, putative)*
13	1200000	1250000	P050, P137	*PKNH_1325500 (CPPUF), PKNH_1325600 (PEPUF), PKNH_1325700 (KAHRP), PKNH_1325800 (PEPUF), PKNH_1325900 (PEPUF), PKNH_1326000 (Plasmodium exported protein, PHIST), PKNH_1326100 (PEPUF), PKNH_1326200 (PEPUF)*
14	1	50000	P050	*PKNH_1400800 (DBPbeta), PKNH_1401000 (PEPUF)*

14	50000	100000	P050	*PKNH_1401100 (PEPUF), PKNH_1401200 (PEPUF), PKNH_1401300 (cytoadherence linked asexual protein, putative), PKNH_1401400 (PEPUF), PKNH_1401500 (lysophospholipase, putative), PKNH_1401600 (PEPUF), PKNH_1401800 (PEPUF), PKNH_1401900 (CPPUF), PKNH_1402000 (PPUF), PKNH_1402100 (PEPUF)*
14	100000	150000	P050	*PKNH_1402200 (PEPUF), PKNH_1402300 (GAP), PKNH_1402400 (ETRAMP), PKNH_1402500 (hypothetical protein), PKNH_1402600 (CPMPUF****)*
14	800000	850000	P137	*PKNH_1417800 (MCM4), PKNH_1417900 (ApiAP2)*
14	3150000	3200000	P050, P137	*PKNH_1472100 (RON3), PKNH_1472200 (CPPUF), PKNH_1472300 (NBPXa)*
14	3200000	3250000	P137	*PKNH_1472400 (tryptophan-rich antigen, putative), PKNH_1472600 (PEPUF), PKNH_1472700 (PEPUF), PKNH_1472800 (Plasmodium exported protei, PHIST), PKNH_1472900 (PEPUF)*

* *Mn-Pk* is the cluster origin for the genetic exchange; ** CPPUF: Conserved *Plasmodium* protein, unknown function; *** PEPUF: *Plasmodium* exported protein, unknown function; **** CPMPUF: Conserved *Plasmodium* membrane protein, unknown function

Supplementary figure 1

Distribution of sequencing coverage across the *P. knowlesi* genome after SWGA

The sequencing coverage for each position was averaged across the 20 high quality isolates undergoing SWGA, after applying quality filtering. A sliding window of 1 kbp was used to improve the clarity of the plot. This plot reveals the uneven coverage across different genetic regions obtained by sequencing SWGA DNA, but shows good representation of genomic positions with >5-fold coverage (dashed horizontal line).

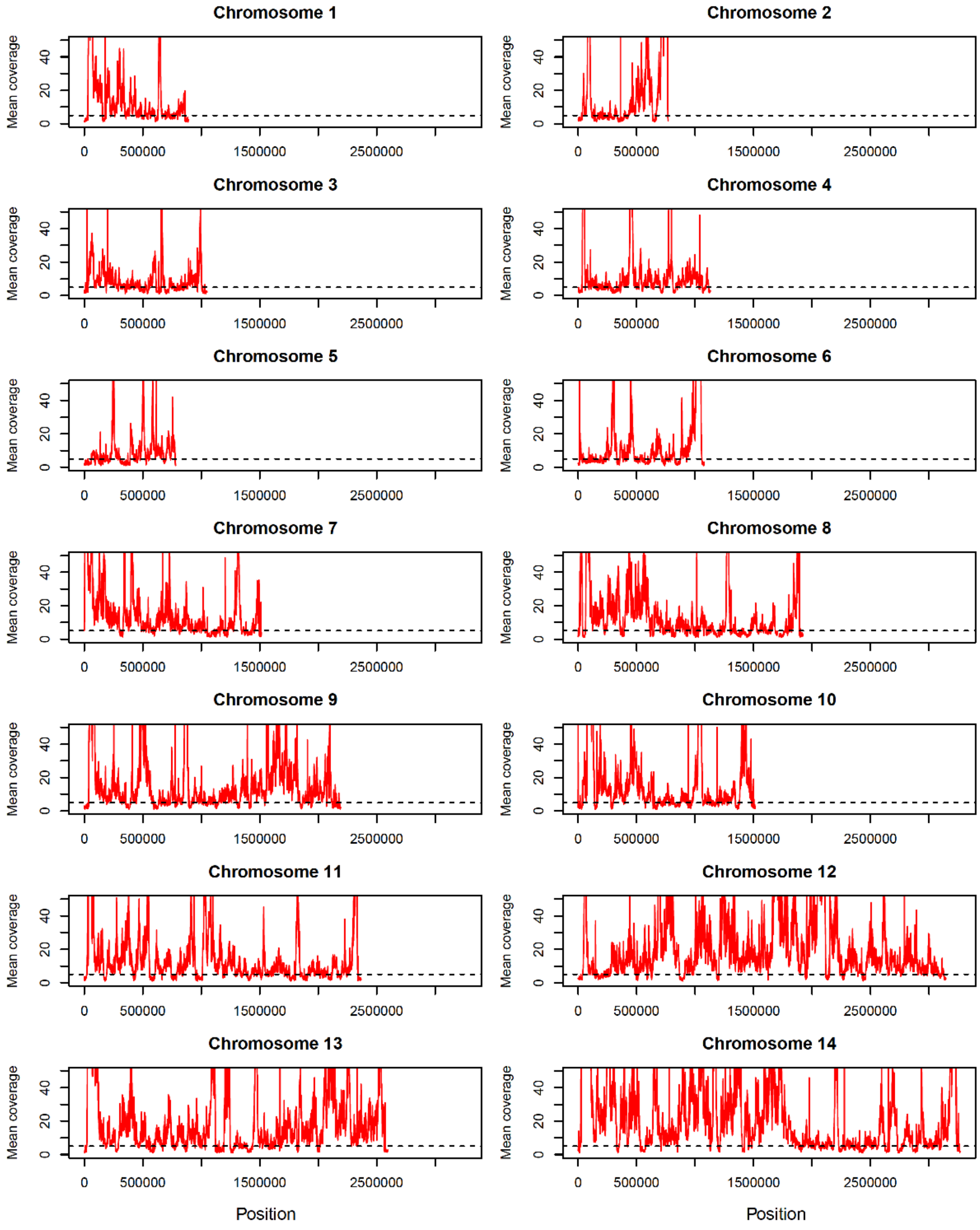

Supplementary figure 2

Estimation of the multiplicity of infection identifies samples with more than one parasite clone

Multiplicity of infection (MOI) was assessed using the overall number of heterozygous calls, as well as the fraction of the genome supporting multiplicity >1 inferred using estMOI software*. This data supports the ability of SWGA to identify mixed infections and suggests no bias towards a specific clone, as samples SAS15 and SAS13 were SWGA amplified and yet have been classified as presenting MOI >1 by both methods. The blue dashed lines highlight the MOI filters applied, where isolates with >15% of genome supporting MOI >1 or > 0.0004% of SNPs with mixed calls, were excluded.

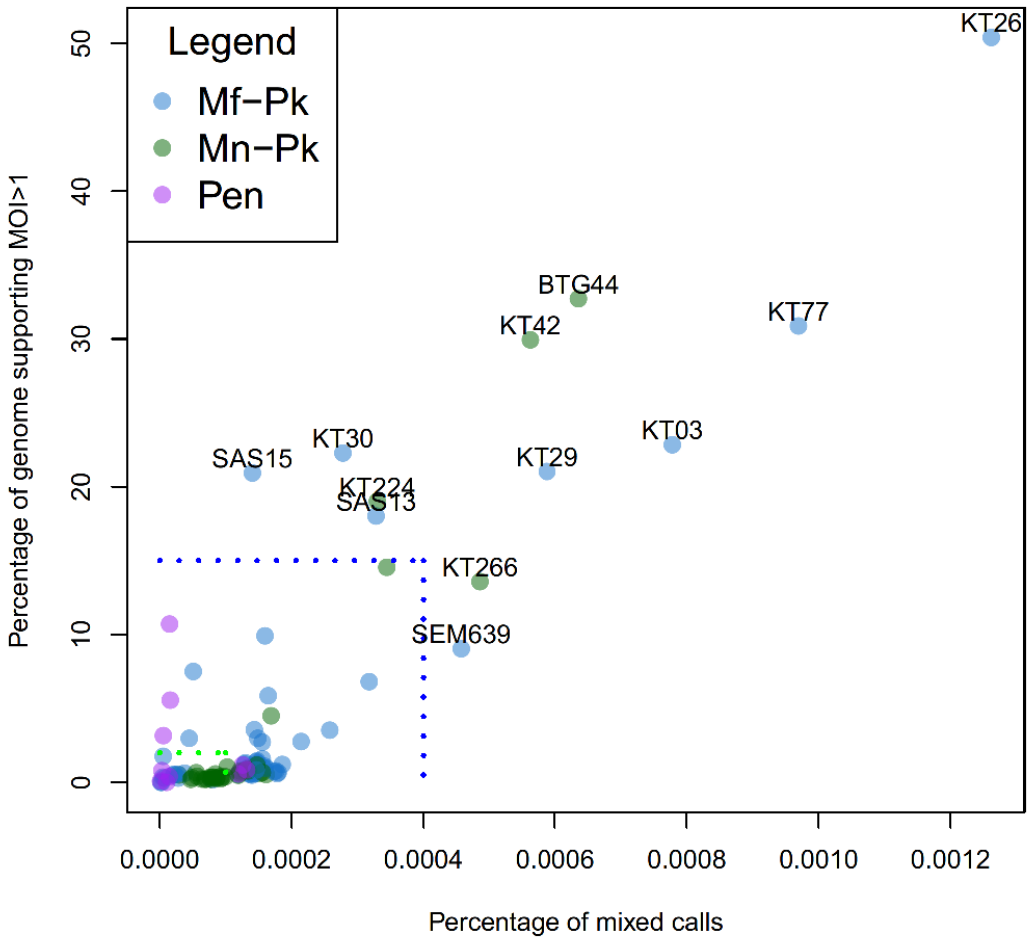

Pen = Peninsular; Borneo Malaysia (*M. nemestrina* (Mn-Pk) and *M. fascicularis* (Mf-Pk) macaques and humans)

* Assefa, S. A. *et al.* estMOI: estimating multiplicity of infection using parasite deep sequencing data. *Bioinformatics* **30**, 1292–1294 (2014).
